# Alcohol consumption among Iranian population based on the findings of STEPS survey 2021

**DOI:** 10.1038/s41598-024-66257-w

**Published:** 2024-07-22

**Authors:** Amirali Hajebi, Maryam Nasserinejad, Negar Rezaei, Sina Azadnajafabad, Mohammad-Mahdi Rashidi, Naser Ahmadi, Erfan Ghasemi, Yosef Farzi, Moein Yoosefi, Shirin Djalalinia, Nima Fattahi, Shahabeddin Rezaei, Elmira Foroutan Mehr, Ameneh Kazemi, Rosa Haghshenas, Kamyar Rezaee, Azadeh Momen Nia Rankohi, Massomeh Afsari, Alireza Mahdavihezaveh, Hamidreza Jamshidi, Farshad Farzadfar

**Affiliations:** 1 Non-Communicable Diseases Research Center, Endocrinology and Metabolism Population Sciences Institute, Tehran University of Medical Sciences, Tehran, Iran; 2 Center for Life Course Health Research, Faculty of Medicine, , University of Oulu, Oulu, Finland; 3 Development of Research and Technology Center, Deputy of Research and Technology Ministry of Health and Medical Education, Tehran, Iran; 4 Human Nutrition Program, Department of Human Sciences, The Ohio State University, Columbus, OH USA; 5 Endocrinology and Metabolism Research Center, Endocrinology and Metabolism Clinical Sciences Institute, Tehran University of Medical Sciences, Tehran, Iran; 6 NCD Management Office, Ministry of Health and Medical Education, Tehran, Iran; 7 Deputy of Health, Ministry of Health and Medical Education, Tehran, Iran; 8 Department of Pharmacology, School of Medicine, Research Institute for Endocrine Sciences, Shahid Beheshti University of Medical Sciences, Tehran, Iran

**Keywords:** Epidemiology, Risk factors, Public health

## Abstract

Alcohol production and consumption have been prohibited in Iran for over four decades, leading to a typical underestimation of its consumption. This study aimed to assess the prevalence of alcohol consumption, its associated factors, and estimate per capita alcohol consumption among Iran’s adult population. In this population-based survey, 27,874 adults from across Iran were selected using systematic proportional-to-size cluster sampling. Alcohol consumption was evaluated through a modified Persian version of the STEPS questionnaires from previous studies, applied over different timespans. Per capita consumption was calculated using the quantity-frequency method, expressed in liters of pure alcohol. Adjusted odds ratios were reported for associates of alcohol consumption concerning metabolic risk factors, sociodemographic elements, and lifestyle variables. The prevalence of lifetime alcohol consumption was 6.9% (95% CI 6.5–7.2) in the adult population, with a notable sex difference (males: 13.7% [95% CI 13–14.4]; females: 1.4% [95% CI 1.1–1.6]). The 12 month prevalence was 3.8% (95% CI 3.6–4.1). For individuals aged 18 and older, the per capita alcohol consumption in Iran was 0.12 L. Factors such as being a lifetime smoker, younger, wealthier, and having 7–12 years of education were significantly linked to higher alcohol consumption. Significant associations were also observed between alcohol consumption and having a history of heart attacks (OR = 2.04, 95% CI 1.44–2.89), and physical injuries (OR = 1.88, 95% CI 1.34–2.64). The estimated lifetime and 12-month prevalence of alcohol use in our study were higher among some of the subpopulations. The findings also revealed a complex relationship between alcohol consumption, behavioral risk factors, and metabolic profiles. Consequently, immediate preventive measures tailored to each factor’s association with alcohol use are recommended.

## Introduction

Globally, the harmful use of alcohol is responsible for 3 million deaths every year, which represents 5.3% of all deaths^1^. The recent Global Burden of Diseases (GBD) study identifies alcohol as the primary risk factor for the 25–49 age group regarding attributable burden^2,3^. Between 2010 and 2019, there was an annualized increase of 0.5% in alcohol use, contributing to a significant rise in the percentage of attributable Disability-Adjusted Life Years (DALYs)^2,4^. Alcohol per capita consumption (APC), the total amount of pure alcohol consumed by individuals^5^, has risen from 5.4 L in 2005 to 6.2 L in 2018 among individuals aged over 15 years worldwide^6,7^. Research indicates a link between alcohol consumption and various non-communicable diseases (NCDs), mental and behavioral disorders, and injuries, including road traffic incidents, drowning, poisoning and falls^8^. Additionally, the consumption of bootleg or adulterated alcohol can lead to methanol poisoning, causing hospitalizations and deaths^9,10^. The harmful use of alcohol also leads to significant social and economic losses to individuals and society^1^.

In Iran, a Muslim-majority country in the Middle East, alcohol production, consumption, and trade are criminalized^11^. Also, there is a social stigma linked to alcohol consumption in this country^12^. These factors contribute to lower APC and prevalence of alcohol use in Iran compared to majority non-Muslim countries^10^. However, they have also delayed comprehensive scientific research and healthcare policy development^13,14^. Despite the alcohol ban, alcoholic beverages have the potential to be illicitly manufactured in residential settings and illegally transported into the country, subsequently being distributed through underground channels^15^. Consequently, there is no official data on the production or sales of alcoholic products^15,16^.

To the best of our knowledge, few studies have measured Iran’s APC, and up-to-date information about alcohol consumption in Iran is limited^9,16–19^. However, the increasing trend of alcohol consumption^20^, alongside its association with various NCDs and mental disorders, necessitates a current and detailed report to formulate appropriate population-wide policies and action plans. This study aims to determine the prevalence of alcohol consumption among Iran's adult population, classified by demographics and socio-economic status, and estimate the APC to assess the pattern of alcohol use in 2021. Understanding these patterns is crucial for health authorities to combat the harmful use of alcohol and its extensive negative impacts.

## Materials and method

The World Health Organization (WHO) initiated the STEPwise Approach to NCD Risk Factor Surveillance (STEPS) in 2000, offering a standardized method for global data collection, processing, and dissemination^21^. STEPS is a large-scale, population-based, sequential cross-sectional study that continuously monitors key risk factors for NCDs. In Iran, eight surveys have been conducted based on the WHO STEPS methodology^22^. The current study is part of the most recent STEPS, carried out in 2021^23^. The data collection phase comprised three steps: first, data were collected using the STEPS questionnaire through participant interviews; second, physical examinations were conducted; and third, laboratory assessments were performed on adults over 25^23^.

### Study population

Data collection began in January 2020 and was partially completed by February 2020, when it was suspended due to the COVID-19 outbreak in Iran. It resumed approximately a year later with COVID-19 safety measures in place, and the remaining data were collected between February and April 2021. A total of 28,520 individuals from urban and rural areas across all 31 provinces of Iran were selected using a multistage clustered probability design to create a nationally representative sample. Of these, 27,874 chose to participate in the first step, 27,745 in the second, and 18,119 in the third. The primary reason for non-response was unwillingness to participate. All participants were at least 18 years old and resided in urban or rural areas of one of the 31 provinces of Iran at the time of data collection. Individuals with mental disorders who might not be able to complete the questionnaires, people whose physical conditions prevented them from participating in an anthropometry measurement, people who were unable to provide laboratory samples, and pregnant women were excluded from the study. After obtaining written informed consent, trained in-home interviewers collected a broad range of information on sociodemographic characteristics, household assets, healthcare utilization, health behaviors, history of metabolic risk factors, anthropometric measures, and laboratory data (only for individuals aged ≥ 25 years).

All of the core questions of the WHO STEPS questionnaire and most of the expanded questions were included in this survey’s questionnaire. Also, due to national concerns and policies for the prevention and control of NCDs, some modified and newly added questions were included in the questionnaire. A team of experts in the fields of epidemiology, population health sciences, health education, medicine, oral health, geriatrics, and nutrition was formed to design and validate STEPS 2021 questionnaires in Iran. The protocol was explained comprehensively, and with further details elsewhere^23^.

### Alcohol consumption assessment

For the alcohol consumption assessment part of the STEPS study, a Persian-translated version of earlier STEPS questionnaires, modified for the Iranian cultural and religious context, was used^23^. Alcohol consumption prevalence, a key variable of this study, was assessed over three timespans: lifetime, past 12 months, and past 30 days. Binge drinking was also measured over the past 12 months and 30 days, using the Bettering the Evaluation and Care of Health (BEACH) definition of consuming six or more standard drinks on one occasion^24^. In line with the STEPS protocol, we used a regular cup (approximately 250-300 cc) as the standard measure for beer and wine, and a smaller cup (about 50 cc) for hard liquors such as vodka, whiskey, tequila, cognac, rum, and their domestic equivalents.

APC, another main variable, is defined as the total amount of alcohol consumed in liters of pure alcohol by the population over 15 years old in a calendar year^25^. As our study included individuals aged 18 and older, we reported the APC for the 18 + age group. Several methods exist to calculate APC^9,26^. The social stigma toward alcohol consumption in Muslim-majority countries and the low prevalence and frequency of alcohol use led to an underestimation of methods such as yesterday and last week^27,28^. As a result, the method that we employed in this study is the Quantity-Frequency (QF) method, which is one of the preferred methods to calculate APC in Muslim-majority countries with very low drinking levels^9^. QF is among the earliest methods to assess alcohol use and is most useful when information about occasional high and low drinking days is not required and time is limited^29^. We employed the below formula of the QF method for calculating APC:

$$Alcohol \;per \;capita \;consumption=F\times Q\times \alpha$$

In this formula, F represents the frequency of alcohol consumption, which we asked questions about in the past 12 months during this study, Q is the amount of alcohol consumed in a typical day of drinking, and α is a constant number that refers to the amount of pure alcohol per alcoholic drink. We used the Iranian Ministry of Health’s routinely used definition of a standard drink, which is 13 g of pure alcohol^9^. APC was calculated by multiplying the estimated number of drinking days in the past 12 months by the daily quantity of drinks and then multiplying the result by 13 to calculate the total alcohol consumed annually in grams.

### Definition of variables

For socioeconomic stratification in this survey, the Wealth Index (WI) was computed based on data regarding household assets obtained from questionnaires. This index was then divided into five levels, ranging from the least wealthy (first quintile) to the most wealthy (fifth quintile). Education levels were recorded in four distinct categories: none, 1–6 years, 7–11 years, and 12 or more years. The living environment of the participants was classified as either urban or rural. The ages of the participants were grouped into seven brackets: 18–24, 25–34, 35–44, 45–54, 55–64, 65–70, and above 70. BMI was calculated using the evaluated height and weight for each individual entered the second step of the study. Obesity was defined as a Body Mass Index (BMI) of more than 30 according to references^30^.

The cardiometabolic conditions and other diseases assessed in this study were identified based on participants' self-reports of having the condition or taking medication for its treatment, as outlined in the questions of the STEPS questionnaire tool. The history of heart attacks variable was investigated via related questions in the questionnaire asking whether the individual had a history of heart attacks in his/her lifetime or not. The physical injuries variable was assessed via the same method and considered positive if the individual reported a history of physical injury(s) during the past 12 months prior to the study.

### Data analysis

Data cleaning adhered to WHO STEPS guidelines^21^, and due to the complex design of the study, statistical analyses were performed using the ‘survey’ package in R software version 4.0.5 to provide weighted estimations based on the most recently available data on the population of Iran, extracted from the Iran Census 2016^23^. We considered p-values below 0.05 as statistically significant. Baseline characteristics were presented using weighted means (standard deviation (SDs)) for continuous variables and numbers (weighted percentages) for categorical variables. To investigate the association between sociodemographic characteristics and alcohol consumption and the association between alcohol consumption and the non-communicable diseases investigated in this study, we used multiple and univariate logistic regression models to estimate odds ratios (ORs) and 95% confidence intervals (CIs). Initially, each variable was analyzed using a bivariate regression model. Subsequently, a multiple model was developed, incorporating multiple variables informed by the results of the bivariate analysis. Bivariate analyses were performed to estimate crude ORs, and all variables with p-value < 0.2 (including age, sex, wealth index, level of education and smoking, and conditions of diabetes, hypertension, hypercholesterolemia, history of heart attack(s), stroke, physical injuries, and obesity) entered the multiple model to calculate adjusted ORs.

### Ethical considerations

All eligible individuals were informed about the study's objectives and methods. Participation was voluntary, with written consent obtained from all participants. Ethical approval was granted by the ethics committee of Tehran University of Medical Sciences (IR.TUMS.NIHR.REC.1398.006). The funding source played no role in the study’s design, data collection, analysis, interpretation, manuscript writing, or decision to submit the paper.

## Results

### Overview

The study included 27,874 adults aged 18 and older (mean age (SD): 45.7 (0.1); 55.4% females). Among these participants, 43.6% had more than 12 years of education, and 75% were urban residents.

### Alcohol consumption scale of Iran’s population

The mean lifetime alcohol consumption prevalence was 6.9% (95% CI 6.5–7.2) for the entire population, 1.4% (95% CI 1.1–1.6) for females, and 13.7% (95% CI 13–14.4) for males. Using the Quantity-Frequency (QF) method, the study calculated the Alcohol Per Capita Consumption (APC) as 0.120 L of pure alcohol (95% CI 0.081–0.159). The 12 month prevalence of alcohol consumption was 3.8% (95% CI 3.6–4.1), with 0.9% for females (95% CI 0.7–1) and 7.5% for males (95% CI 7–8.1). In the past 30 days, 2.1% of participants (95% CI 1.9–2.2) reported alcohol consumption, with 0.5% females (95% CI 0.3–0.6) and 4% males (95% CI 3.6–4.4). The overall prevalence of binge drinking in the past 12 months was 1.7% (95% CI 1.5–1.8) (Table 1). Males were nearly four times more likely to consume alcohol than females (OR 3.8, 95% CI 3–4.9) (Table 2). The highest alcohol consumption rates, including binge drinking, were observed in the 18–24 and 25–34 age groups, decreasing with age. In all age groups, males had higher prevalence rates than females (Table 1).

**Table 1:** Alcohol consumption in Iran status and socio-demographic characteristics, based on Iran STEPS 2021 Survey.

		Alcohol drinking status				
		Lifetime alcohol drinking (%)	Past 12 months alcohol drinking (%)	Past 30 days alcohol drinking (%)	Past 12 months binge drinking (%)	Past 30 days binge drinking (%)
Variables	Categories					
Sex	Female	177 (1.4%)	108 (0.9%)	55 (0.5%)	27 (0.3%)	15 (0.1%)
	Male	1593 (13.7%)	869 (7.5%)	460 (4%)	372 (3.4%)	213 (1.9%)
Age	18–24 years	219 (9.19%)	164 (7%)	86 (3.6%)	91 (4.3%)	43 (1.9%)
	25–34 years	501 (10.2%)	326 (6.8%)	176 (3.8%)	148 (3.3%)	89 (2%)
	35–44 years	440 (7.5%)	253 (4.4%)	135 (2.4%)	95 (1.6%)	59 (1%)
	45–54 years	256 (5.1%)	116 (2.3%)	56 (1.1%)	32 (0.7%)	18 (0.4%)
	55–64 years	196 (4.9%)	80 (2.1%)	43 (1.1%)	22 (0.6%)	12 (0.3%)
	65–70 years	112 (4.6%)	29 (1.1%)	15 (0.5%)	9 (0.3%)	6 (0.2%)
	> 70	46 (5%)	9 (1.2%)	4 (0.5%)	2 (0.3%)	1 (0.1%)
Residential status	Rural	456 (6.6%)	247 (3.5%)	134 (1.9%)	92 (1.4%)	55 (0.8%)
	Urban	1314 (7%)	730 (3.9%)	381 (2.1%)	307 (1.8%)	173 (0.9%)
Wealth index	Poor	281 (5.6%)	125 (2.5%)	69 (1.3%)	48 (1%)	29 (0.5%)
	Second quintile	320 (6.2%)	165 (3.2%)	79 (1.4%)	73 (1.6%)	35 (0.7%)
	Third quintile	359 (7.4%)	195 (4.1%)	107 (2.3%)	84 (1.8%)	45 (1%)
	Forth quintile	360 (7.8%)	213 (4.6%)	107 (2.5%)	82 (1.9%)	45 (1%)
	Rich	388 (8.1%)	236 (5%)	131 (2.8%)	96 (2.2%)	62 (1.4%)
Lifetime smoking	No	1122 (4.7%)	615 (2.6%)	312 (1.4%)	235 (1.1%)	131 (0.6%)
	Yes	647 (27.6%)	361 (16%)	203 (8.9%)	164 (7.4%)	97 (4.2%)
Total		1770 (6.9%)	977 (3.8%)	515 (2.1%)	399 (1.7%)	228 (1.2%)

**Table 2:** Assessment of lifetime alcohol consumption associated factors using multiple logistic regression.

		Lifetime alcohol consumption			
Variable	Category	OR (95% CI) Model a^1^	P value	OR (95% CI) Model b^2^	P value
Sex	Female	Ref	–	–	–
	Male	11.56 (9.74,13.71)	< 0.001	3.84 (2.99,4.94)	< 0.001
Residential status	Rural	Ref	–	–	–
	Urban	1.06 (0.94,1.20)	0.32	–	–
Wealth index	Poor	ref	–	–	–
	Second quintile	1.1 (0.91,1.32)	0.32	1.07 (0.85,1.35)	0.57
	Third quintile	1.34 (1.12,1.60)	0.001	1.26 (1.01,1.57)	0.04
	Forth quintile	1.41 (1.19,1.69)	< 0.001	1.34 (1.07,1.67)	0.01
	Rich	1.47 (1.23,1.75)	< 0.001	1.49 (1.18,1.87)	0.001
Education	Illiterate	Ref	–	–	–
	Less than 7 years of education	3.08 (2.29,4.12)	< 0.001	1.58 (1.12,2.22)	0.01
	7–12 years of education	6.33 (4.76,8.41)	< 0.001	2.23 (1.58,3.15)	< 0.001
	More than 12 years of education	4.38 (3.32,5.78)	< 0.001	1.67 (1.18,2.36)	0.01
Lifetime smoking	No	Ref			
	Yes	7.65 (6.80,8.62)	< 0.001	4.17 (3.58,4.85)	< 0.001

^1^Crude odds ratio.

^2^Adjusted odds ratio (all variables with p-value < 0.2 entered the model including age, sex, wealth index, level of education and smoking).

### Associates of alcohol consumption

**Figure 1:**
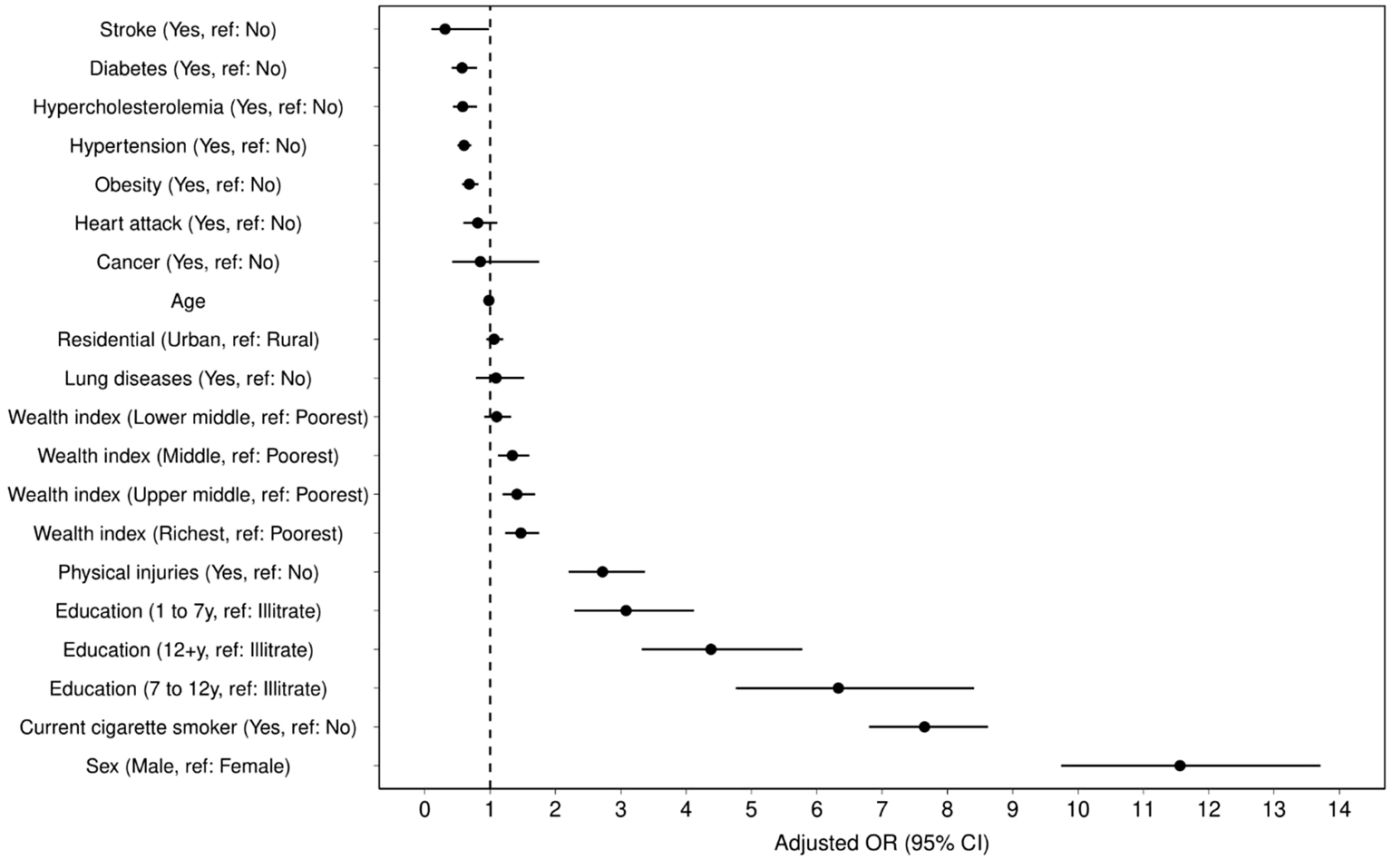
Forest plot of lifetime alcohol consumption correlates and associated metabolic diseases and risk factors analyzed by adjusted ORs.

Table 2 shows the crude and adjusted odds ratios for sex-categorized variables. Generally, the adjusted odds for lifetime alcohol consumption were significantly higher among males (OR = 3.84, 95% CI 2.99–4.94), smokers (OR = 4.17, 95% CI 3.58–4.85), and the wealthiest compared to the poorest (OR = 1.49, 95% CI 1.18–1.87) (Fig. 1). Also, it has been shown that the adjusted odds of lifetime alcohol consumption significantly decrease with age (OR = 0.98, 95% CI 0.97–0.98). Individuals with 7–12 years of education had the highest odds of alcohol consumption (OR = 2.23, 95% CI 1.58–3.15).

Multivariable logistic regression revealed that individuals reporting lifetime alcohol consumption had significantly greater odds of having a history of heart attacks (OR = 2.04, 95% CI 1.44–2.89), and physical injuries (OR = 1.88, 95% CI 1.34–2.64). The study also found positive associations between lifetime alcohol consumption and certain metabolic diseases and risk factors, but negative associations with stroke and diabetes. However, these associations were not significant (Table 3, Fig. 1).

**Table 3:** Assessment of alcohol consumption association with metabolic diseases and risk factors using multiple logistic regression.

		Life time alcohol consumption			
Variable	Category	OR (95% CI) Model a^1^	P value	OR (95% CI) Model b^2^	P value
Diabetes	No	Ref	–	–	–
	Yes	0.57 (0.41,0.80)	0.001	0.80 (0.57,1.11)	0.18
Hypertension	No	Ref	–	–	–
	Yes	0.60 (0.50,0.71)	< 0.001	1.06 (0.83,1.35)	0.64
Hypercholesterolemia	No	Ref	–		–
	Yes	0.58 (0.43,0.80)	0.001	1.13 (0.83,1.55)	0.44
History of heart attack(s)	No	Ref	–	–	–
	Yes	0.81 (0.59,1.11)	0.18	2.04 (1.44,2.89)	< 0.001
Stroke	No	Ref	–	–	–
	Yes	0.31 (0.10,0.98)	0.04	0.79 (0.38,1.66)	0.54
Cancer	No	Ref	–	–	–
	Yes	0.85(0.42,1.75)	0.67	–	–
Lung diseases	No	Ref	–	–	–
	Yes	1.09 (0.78,1.52)	0.61	–	–
Physical injuries	No	Ref	–	–	–
	Yes	2.72 (2.20,3.37)	< 0.001	1.88 (1.34,2.64)	< 0.001
Obesity	No	Ref	–	–	–
	Yes	0.68 (0.57,0.82)	< 0.001	1.23 (0.97,1.56)	0.01

^1^Crude odds ratio.

^2^Adjusted odds ratio (all variables with p-value < 0.2 entered the model including age, sex, wealth index, level of education and smoking, and conditions of diabetes, hypertension, hypercholesterolemia, history of heart attack(s), stroke, physical injuries, and obesity).

## Discussion

This study evaluated alcohol consumption prevalence among Iranians aged 18 and older. The findings indicate that approximately 3.9 million and 2.2 million individuals over 18 in Iran have consumed alcohol at least once in their lifetime and in the past 12 months, respectively.

The lifetime and 12 month prevalence rates of alcohol consumption in our study are lower than the global average but higher than the figures estimated by the WHO for EMR countries^6^. It’s important to note that the WHO’s estimates refer to populations aged over 15 years. Additionally, our study showed a lower lifetime prevalence of alcohol consumption than that reported in four Iranian provinces in 2015^15^. Part of this difference can be attributed to the setting of the studies. The 2015 study was a street-based study, and the participants faced less stigma when disclosing facts about their alcohol consumption. Essentially, the prohibition of alcohol consumption in Iran and other Muslim-majority countries usually leads to under-reporting of alcohol use^17,31^. Our study likely reflects this difference, which is due to underreporting and suggests that the actual prevalence may be higher than reported. Compared to other countries, the prevalence of binge drinking in the last month and last 12 months was notably lower in our study^32–34^.

A higher prevalence of alcohol consumption was observed in male participants compared to females, aligning with both national^9,15,35^, and international studies^6,36,37^. This gender disparity in alcohol consumption could be attributed to cultural and religious norms, as women typically adhere more closely to cultural and religious principles that discourage alcohol use^37^. Consistent with other research, our study found that binge drinking was more prevalent among males^38–41^.

This study indicates that the highest prevalence of alcohol consumption in the past month, past 12 months, and over a lifetime occurred in the 18–34 age group for both sexes. The higher prevalence of alcohol consumption in this age group is likely influenced by various developmental and cultural factors^42^. Young adults are prone to consuming larger quantities of alcohol, leading to more adverse effects, and individuals between 20 and 34 years old experience the highest mortality rates related to alcohol consumption^43^.

The results also demonstrated that alcohol consumption prevalence is higher among cigarette smokers across all age groups. Correspondingly, higher instances of binge drinking were noted among smokers, aligning with research showing a link between tobacco and alcohol use^44^.

Moreover, a greater prevalence of alcohol consumption was observed among urban residents and individuals with higher education levels, highlighting the impact of social factors, living conditions, and education on alcohol consumption in Iran. Studies within Iran have shown similar patterns, with higher alcohol consumption among high school and university students^45–47^. These findings are consistent with results from other countries^48–50^. Research on the geographical relationship with alcohol consumption presents mixed results; some studies report higher consumption in urban areas^51^, while others find increased consumption in rural settings^52^.

In our study, individuals with a higher wealth index exhibited increased alcohol consumption, with the wealthiest group consuming 1.5 times more than those in the lowest wealth index category. Numerous studies have established a direct association between socio-economic status and alcohol consumption, yet an inverse relationship exists between harmful drinking patterns, such as binge drinking, and socio-economic status^53–58^.

Research indicates that in societies where alcohol consumption is stigmatized due to legal and religious prohibitions, as well as criminalization, there is a tendency for alcohol users to underreport their usage. This underreporting is particularly pronounced when questions pertain to recent consumption (past day or week), while queries about the past month or year tend to yield more accurate results. In contrast, in countries without such prohibitions, questioning about last day or week alcohol consumption tends to be more reliable, effectively addressing recall bias and serving as a better tool for calculating APC^9,28,59^. Given the legal and social stigma associated with alcohol in Iran, our survey considered the most recent recall period as the last month. The Quantity-Frequency (QF) method was used for annual APC calculation, although it entails more recall bias than the “last week” method^60^. The survey results showed that Iran’s APC (0.120L) is lower than the global (6.4L) and regional (0.6L) averages^6^. It’s important to note that while these international figures typically pertain to individuals over 15 years old, our study focused on those over 18, which might contribute to some of the observed differences. Compared to the QF calculation by Amin-Esmaeili et al. in 2011, the APC reported in this study showed a 50% increase^9^.

The results from the multiple logistic regression model in our study indicated a significant positive correlation between alcohol consumption and various non-communicable diseases and metabolic syndromes, including hypertension, hypercholesterolemia, heart attacks, obesity, and physical injuries. Interestingly, the data suggested a decrease in the risk of diabetes mellitus and stroke associated with alcohol consumption. Particularly noteworthy is the elevated and significant risk linked with heart attacks, obesity, and physical injuries. Various studies have explored the relationship between alcohol consumption and non-communicable diseases. Identified patterns include the J-shaped curve, illustrating a dose–response relationship between alcohol intake and the odds of metabolic syndrome^61^, and the U-shaped pattern, where heavy drinkers face a significantly higher risk, unlike low and moderate consumers^62^. While some studies suggest a linear relationship between alcohol consumption and metabolic syndrome^63,64^, others find no significant correlation^65,66^. The growing body of research underscoring the link between lifetime alcohol consumption and its detrimental effects, especially the risk of metabolic syndrome, highlights the urgent need for preventive measures against alcohol consumption^3,67,68^.

Despite the challenges posed by the COVID-19 pandemic, this study's data collection was conducted face-to-face, adhering to health guidelines and social distancing. This national study encompasses a large sample size from across the country. A key feature of this study is its alignment with the WHO’s STEPS methodology, enabling comparisons with global studies. In response to the stigma surrounding alcohol consumption in Iran, interviewers were specifically trained to gather data discreetly and privately. Another strength of this study lies in its assessment of comorbidities associated with alcohol consumption. Conditions like hypercholesterolemia and diabetes were evaluated using both laboratory data and medical history, and participants' blood pressure was measured during their assessment.

This study is subject to some limitations. Firstly, its cross-sectional design poses challenges in identifying potential reverse associations between alcohol consumption and assessed outcomes, as well as in establishing causality. As a household survey, it does not include individuals living in residential facilities, institutions with restricted access, or those who are homeless, where alcohol consumption prevalence might differ. Additionally, the reliance on self-reporting could lead to underreporting, particularly since alcohol consumption is illegal and socially stigmatized in Iran. This study focuses on individuals over 18 years old, which limits the comparability of the Alcohol Per Capita Consumption (APC) results with other national and international studies that often include those aged 15 and older. Furthermore, restricted access to recent alcohol consumption data (last week) made it impossible to employ the “last week” method alongside the Quantity-Frequency (QF) method. Consequently, calculating APC using a combination method was not feasible^9^. A key limitation of our study is the low prevalence of alcohol consumption in the Iranian population, potentially due to under-reporting or genuinely lower consumption levels. This led to smaller sample sizes for recent consumption periods (past 12 months and 30 days), limiting the power and reliability of our statistical analyses. The impact of COVID-19 on changes in alcohol consumption levels could be significant during the pandemic based on the literature, but measuring this impact was beyond the scope of our study, and it was another limitation of the current research.

## Conclusion

In this population-based study, we estimated the prevalence of alcohol consumption over multiple timespans, finding the highest rates among males and younger age groups. This underscores the need for targeted prevention strategies within these demographics in public health policies. The potential hazardous effects of even lifetime alcohol consumption call for appropriate preventive measures to address this public health concern. Moreover, focusing on harm reduction interventions among those with lower socioeconomic status is crucial to mitigate the adverse impacts of harmful alcohol use. Our findings also highlight a positive correlation between alcohol consumption and metabolic syndrome. To reduce the possible negative effects of alcohol on individuals' metabolic profiles, implementing preventive strategies against alcohol consumption, particularly its harmful patterns, is a critical priority.

## Data Availability

The datasets used and/or analyzed during the current study are available from the corresponding author on reasonable request.
